# 

**Published:** 1905-07

**Authors:** 


### INDEX TO VOLUME XXI.

A Bomb Under the Octopus ..................................361
Abdominal Section for Fecal Impaction. By John M. Neel, M. D., Bonham, Texas..................................161
Abscess of the Female Pelvic Organs. By J. P. Oliver, M. D., Caldwell, Texas ..................................123
Abstracts and Selections........65, 106, 141, 177, 209, 252, 293, 337, 369..................................448
A Case of Early Menopause. By Emory Lanphear, M. D., Ph. D., LL. D., St. Louis, Mo..................................437
American International Congress on Tuberculosis, 1906......327
American International Congress on Tuberculosis............444
A Note on the Improper Management of Epileptics. By Marc Ray Hughes, M. D., St. Louis, Mo......................443
A Petrified Lens and An Ossified Chorion Occurring in the Same Eyeball. By Sam R. Burroughs, M. D., Buffalo, Texas ..................................115
A Rare Treat..................................132
A Tribute to Doctors. By Carrie Belle Sterrett.............324
Bronchitis and Broncho-Pneumonia in Children. By Wm. A. Wood, M. D., Gallatin, Mo..................................321
Books and Magazines ..................................
............26, 69, 110, 145, 184, 218, 256, 302, 345, 418, 463
Dr. Simmons and the Octopus Again ......................205
Ectopic Pregnancy. By Henry K. Leake, M. D., Dallas, Texas ..................................325
Editorialets..........135, 174, 207, 251, 289, 334, 367, 409, 445
Gross Abnormalities of the Appendix Vermiformis Noted in 3550 Autopsies. By A. P. Heineck, M. D., Chicago, Ill....313
Harpooning the Devil Fish.................................. 19
Hemoptysis, Causes and Treatment. By L. Sexton, M. D., New Orleans, La. ..................................167
Homeopathy to the Rescue.................................. 99
Human Hair in the Stomach. By S. S. Rather, M. D., Jacksonville, Texas ..................................358
Intestinal Obstruction, With Special Reference to the Duty of the General Practitioner. By W. C. Lipscomb, M. D., Crockett, Texas .................................. 10
Letter from Dr. Stroud, of Terrell, Texas.................. 48
Memorial Address. By F. E. Daniel, M. D., Austin, Texas....351

Mosquito Extermination and Its Bearing on the Yellow Fever and Modern Quarantines. By C. W. Trueheart, M. D., Galveston, City Health Officer....391
Nervous Dyspepsia (Neurasthenia Gastrica)—Its Diagnosis and Treatment With Report of Cases. By John T. Moore, M. D., Galveston, Texas....233
News and Miscellany....23, 61, 104
Notes from "The Mayo Clinic".... 45
Obstetric Practice in China....359
Our Visitors....138
Peculiar Gin Accident. By J. P. Oliver, M. D., Caldwell, Texas....198
Pneumonia....139
Publisher's Notes....32, 74, 112, 150, 192, 226, 257, 307, 348, 469
Puerperal Eclampsia. By T. B. Taylor, M. D., Elgin, Texas..118
Pulmonary Consumption, Bright's Disease and Diabetes Mellitus. By B. F. Felchel, M. D., Chicago, Ill....434
Quackery. By Dudley F. Sicher, Yale University....273
Report of a Case of Splenectomy. By D. L. Peeples, M. D.. Navasota, Texas.... 8
Retiring Address. By C. M. Harrison, M. D., Cooper, Texas.. 85
Sanitary Convention at Austin....394
Society Notes....98, 239
Some Remarks on Fracture of the Head of the Femur. By T. J. Bennett, M. D., Austin, Texas.... 41
State Medical Association of Texas....403
Status of the Colored Brother in Relation to the A. M. A.....362
Surgeons and Damage Suits. By J. W. Kenney, M. D., San Antonio, Texas....195
Texas Escapes Epidemic....173
The American International Congress on Tuberculosis....401
The Conceit of the Reformers....287
The Doctor's Wife. By V. A. H. D.... 13
The Drug Drummer's Story.... 53
The Effect of Lesions of the Oral Cavity Upon Other Organs and the System at Large and the Relation of the Physician Thereto. By Price Cheaney, M. D., D. D. S., Dallas, Texas..155
The Evolution of Medicine. By Malone Duggan, M. D., San Antonio, Texas.... 77
The Great American Blight....129
The Incredibility of Veracity....241

The Medical Journal Trust and the Independent Medical Press . . . . . . . . . . . . . . . . . . . . . . . . . . . . . . . . . . . . . . . . . . . 15
The New Pharmacopeia. By R. P. Daniel, San Antonio, Texas . . . . . . . . . . . . . . . . . . . . . . . . . . . . . . . . . . . . . . . . . . . 93
The Passing of Quarantine. . . . . . . . . . . . . . . . . . . . . . . . . . . . . .203
The Relation of the Physician and the Druggist. By F. S. White, M. D., Terrell, Texas. . . . . . . . . . . . . . . . . . . . . . . . . . .281
The State Medical Association's Efforts at Medical Legislation.439
The Treatment of Incipient Insanity. By A. D. Young, M. D., Oklahoma City, Okla. . . . . . . . . . . . . . . . . . . . . . . . . . . . . . . . .285
Tripartite Mentality. By J. T. Searcy, M. D., Tuscaloosa, Ala. . . . . . . . . . . . . . . . . . . . . . . . . . . . . . . . . . . . . . . . . . . . 1
Two Remarkable Cases of Intestinal Perforation. By Emory Lanphear, M. D., Ph. D., LL. D., St. Louis Mo. . . . . . . . . . . .200
Valyl. By A. Ammelburg. . . . . . . . . . . . . . . . . . . . . . . . . . . . . . .163
War on the Vampire; a Layman in the Lead. . . . . . . . . . . . . . . .327

